# Combating Bovine Mastitis in the Dairy Sector in an Era of Antimicrobial Resistance: Ethno-veterinary Medicinal Option as a Viable Alternative Approach

**DOI:** 10.3389/fvets.2022.800322

**Published:** 2022-04-04

**Authors:** Daniel Jesuwenu Ajose, Bukola Opeyemi Oluwarinde, Tesleem Olatunde Abolarinwa, Justine Fri, Kotsoana Peter Montso, Omolola Esther Fayemi, Adeyemi Oladapo Aremu, Collins Njie Ateba

**Affiliations:** ^1^Food Security and Safety Niche Area, Faculty of Agriculture, Science and Technology, North-West University, Mmabatho, South Africa; ^2^Department of Chemistry, Faculty of Natural and Agricultural Sciences, North-West University, Mmabatho, South Africa; ^3^Indigenous Knowledge Systems (IKS) Centre, Faculty of Natural and Agricultural Sciences, North-West University, Mmabatho, South Africa

**Keywords:** mastitis, ethnobotany, antibiotics, milk output, infectious conditions

## Abstract

Bovine mastitis (BM) is the traditional infectious condition in reared cattle which may result in serious repercussions ranging from animal welfare to economic issues. Owing to the high costs associated with preventative practices and therapeutic measures, lower milk output, and early culling, bovine mastitis is accountable for most of the financial losses suffered in cattle farming. *Streptococcus agalactiae, Staphylococcus aureus, Streptococcus dysgalactiae* and coliform bacteria are the predominant pathogens for bovine mastitis. In addition, the occurrence of BM has been linked to lactation stage and poor management, in the latter case, the poor stabling conditions around udder hygiene. BM occurs throughout the world, with varying rates of *Streptococcus agalactiae* infection in different regions. Despite the modern techniques, such as the appropriate milking practices that are applied, lower levels of pathogen vulnerability may help to prevent the development of the disease, BM treatment is primarily reliant on antibiotics for both prophylactic and therapeutic purposes. Nevertheless, as a result of the proliferation of bacterial agents to withstand the antibiotic effects, these therapies have frequently proven ineffectual, resulting in persistent BM. Consequently, alternative medicines for the management of udder inflammation have been researched, notably natural compounds derived from plants. This review focuses on BM in terms of its risk factors, pathogenesis, management, the molecular identification of causative agents, as well as the application of ethno-veterinary medicine as an alternative therapy.

## Introduction

**Figure 1:**
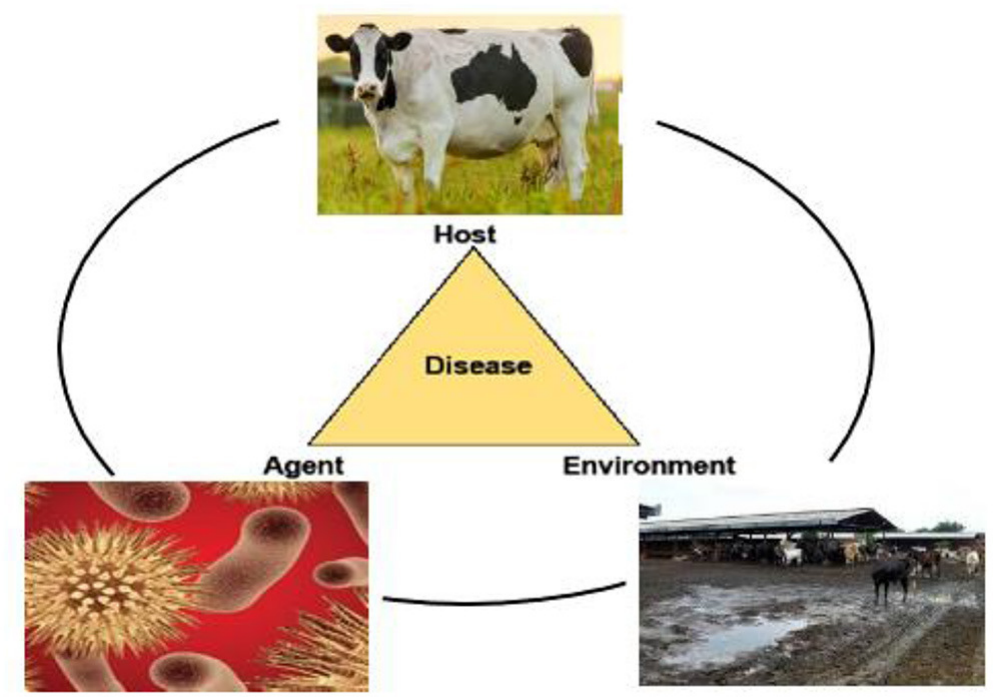
Factors contributing to bovine mastitis.

Bovine mastitis (BM) is associated with the swelling of the udder which is caused by various factors, such as contagious pathogens, poor nutrition, and ineffectual management conditions on the farm (1, 2) (Figure 1). Its effects range from inferior animal welfare to mediocre industrial economic conditions, some of which encompass, without bounds, reduced milk yield, increased therapeutic costs, and premature culling (3–5). Moreover, different microorganisms, including bacteria, can cause mastitis. Pathogens that are most frequently encountered in a mastitic condition are *Staphylococcus aureus, Streptococcus uberis, Streptococcus agalactiae, Streptococcus dysgalactiae, Escherichia coli*, and other coliforms (6). Vanderhaeghen et al. (7) state that the clinical presentation of bovine mastitis assumes two distinct forms (clinical or subclinical) (8), which are distinguished by a rise in the pathological cell number. Clinical mastitis is marked by evident alterations in the quality and composition of the milk, including the level of coagulants, and is often followed by pervasive indicators and impairments, *inter alia* high temperatures, in the animal. On the other hand, BM caused by environmental pathogens is characterized by an increase in the somatic cell count (SCC), resulting in significant cost implications, especially on account of the increased cell count in milk samples (2, 8). According to Giesecke et al. (9), annual milk wastage owing to BM on farms in South Africa amounted to approximately ZAR 29.68 million. More recent data on the prevalence of BM in South Africa are unavailable, probably due to the fact that the condition is not commonly and regularly reported (10).

Antibiotics have long been considered the first line of defense against bacterial infections in dairy cattle, especially in the case of mastitis, where antibiotic residues occur in the milk and there is the risk that microbial resistance will spread to the environment (5, 11). Owing to the proliferation of multiple antibiotic-resistant (MAR) bacteria, which is a pressing public health concern for animal and human health, food security, and development, the use of antibiotics in animal production has been studied with great caution (12, 13). From the one-health perspective, mastitis-causing bacteria have broken through a number of hierarchical barriers, allowing for zoonotic transmission from bovines to people *via* milk and meat, thus putting public health at risk. Hence, there is a significant risk of microbial contaminants from the animal to the pastures and the equipment, to the effect that the consumption of raw milk may cause food-borne infections. Pasteurization of milk is thus essential to guarantee its quality and to extend its shelf-life (14).

In this age of the proliferation of microbial resistance to antimicrobials, it is imperative to source alternative medicines for the management and prevention of bovine mastitis. Hence, this review focuses on BM in terms of risk factors, pathogenesis, control and treatments, the molecular identification of causative agents, as well as the application of ethno-veterinary medicine (EVM) as an alternative therapy for BM.

## Method for Selection of Literature

Keywords such as bovine mastitis, ethno-veterinary medicine, livestock and antimicrobial resistance were used to promote the literature search technique. In addition, phrases such as ethno-botany, alternative medicine, anti-inflammatory and antibacterial effect were employed to generate data for the biological activity and the phytochemical characteristics discussed in the review. These keywords were used alone and in combination to find relevant material from electronic data base such as Google Scholar, Pubmed, Science Direct, and Web of Science. To be considered for inclusion in the review, an article or study should have included and identified information about the usage of a specific medicinal plant in the ethno-medicinal context for treating BM within the research period (i.e., up until October 2021).

The following data were obtained for each study: the plant's scientific name, plant part, life form, disease treated, dose, preparation and administration. Articles reporting on the ethno-veterinary applications (**Table 2**) and the preparation and administration of medicinal plant products against antimicrobial resistant BM pathogens (**Table 3**) met the inclusion criteria. Review and research papers not involving the ethno-veterinary medicinal approach in combating antimicrobial resistance of mastitis pathogens were excluded. Finally, all papers that met the inclusion criteria were retrieved for the collection of adequate information. All scientific plant names were cross-referenced on the international databases, namely The World Flora Online (http://www.worldfloraonline.org/) and The Plant List (www.theplantlist.org).

## Burden of Mastitis on Dairy Farms

Udder inflammation is often regarded as the ultimate threat to the dairy sector, resulting in both financial losses and harmful impacts on public health. Mastitis is also the foremost source of mortality in dairy cows. Mastitis-related financial losses are difficult to quantify; however, financial losses are attributed to the cost of medications, veterinary services, laboratory fees, and additional labor for the farmer. Losses also include those incurred on account of lost milk, culling/deaths and treatment costs (29). Amuta et al. (30) reported that the majority of farmers covered in their study in the North-West region of Nigeria considered clinical mastitis (CM)-related milk production losses to be significant (55%).

In Germany, milk wastage, ranging from 27 to 52%, (median rate; 37%) and rejected milk, ranging from 13 to 30%, (median value; 20%), were the most expensive charges associated with mastitis (29). Replacement expenditures, which were due to culling, and mastitis mortality, ranging from 3 to 28% with a median value of 22%, were the next biggest losses reported (median 22%). More so, Beyene and Tolosa (31) reported an average yearly drop of 59,719.08ETB, equivalent to USD 2,949.8, on account of mastitis, and a milk loss of 22.3% in crossbred cattle and of 2.24% in local zebu cattle in Ethiopia. The productive impact of subclinical mastitis in Colombian cattle ranches was estimated at about USD 800,000 per year, with milk production losses ranging from 1.3 to 13.5% per farm (32).

The losses in the value of milk and veterinary expenses in Central India on account of mastitis were reported to be about 49 and 37%, respectively (33). According to a study released by the University of Glasgow (https://www.fortunebusinessinsights.com/bovine-mastitis-market-103482), mastitis is predicted to cost the global dairy industry between USD 19.7 and 30 billion every year. Furthermore, another associated cost is a shortening of the lactation term of the animals. According to Khan and Khan (34), each infected animal's lactation period is reduced by about 57 days, while Seegers et al. (35) described reductions in mean milk output to be 375 kg for each lactation.

## Risk Factors and Prevalence of Bovine Mastitis

The harmful factors associated with BM are classified as intrinsic and extrinsic and include, amongst others, species, age, parity, suckling period, antiquity of condition, state of habitat, and common handling conditions (36). These were all found to be remarkably related to BM. Conversely, milk-gland washing and drying and antiquity of condition had no remarkable outcome on the occurrence of BM (37). Nearly 30% of Saharan countries currently register instances of BM, leaving the rest of the continent in the dark (38). At the cow stage, the overall mastitis prevalence recorded on dairy farms in some parts of Africa is < 54%, with CM at < 14% and subclinical mastitis at 16 to 88%. Mastitis was found to be more than 50% prevalent in previous research studies conducted in various parts of the southern region of Saharan Africa, with subclinical mastitis ranging from 16 to 80% (39). Mastitis is also common in Asian countries, with a prevalence of over 50% (40). Kvapilik et al. (41) published the global prevalence of sub-CM as ranging from 15 to 75%. Also, with the increase in the counts of cattle farms, the frequency of BM is escalating tremendously on a yearly basis.

## The Pathogenesis and Prevalence of Bovine Mastitis

Invading pathogens penetrate the irritated udder through the teat canal, which is located at the anterior margin of the cow mamilla (42, 43). In addition to physical stress and chemical irritants, bacterial pathogens grow and produce toxins that cause harm to the milk-producing tissue. The quantity of leukocytes in the milk increases as an effect of these factors. The initial line of defense is presented by the bovine mammary epithelial cells (bMECs). Thus, the bMECs play a significant role in that they provide broad and unique protection against resistant pathogenic microorganisms (42). More so, the udder may be contaminated by a number of microbial pathogens, which kill these cells and result in mastitis (44, 45).

A sphincter of smooth muscles covers the mamilla of the milk duct and works to ensure that it is closed from the outside (46). By preventing milk from escaping, it also prevents pathogens from entering the teat. Keratin generated from the stratified squamous epithelium lines the mamillary duct from the inside. The effectiveness of the keratin in the teat canal is to suppress the bacteria to a limited extent and to prevent their proliferation (47). Keratin is a waxy substance made up of phospholipids and fibrous proteins. Keratin fiber proteins are able to bind electrostatically to mastitis pathogens in the teat canal, thus altering the bacterial cell wall and making it more vulnerable to osmotic pressure. Invading pathogens are inhibited and killed when osmotic pressure is not maintained (48). During the milking process, germs near the teat's opening find a way into the teat canal, causing shock and damage to the keratin and mucous membranes surrounding the teat sinus (49). Also, after milking, the teat canal may remain partially open for 1 to 2 h, allowing infections to freely enter the teat canal (50).

The global prevalence rates of subclinical (SCM) and clinical mastitis (CM) are 42 and 15%, respectively (51). The SCM prevalence was found to be higher when compared to CM prevalence in the countries of the World. In most of the studies, CMT and its clinical manifestation were used for diagnosing SCM and CM, respectively. The pervasiveness rates for SCM and CM were found to be 45 and 18%, respectively in India. As stated by the College of Veterinary Medicine, Cornell University, clinical mastitis affects from 0 to 200 cows per 100 annually. Several studies from around the world have estimated the yearly occurrence of clinical mastitis as 25 to 30 cases per 100 cows (https://www.fortunebusinessinsights.com/bovine-mastitis-market-103482). In an Ethiopian study, the overall mastitis occurrence was reported to be 73.7% (283/384), of which 28.9% (82/283) was of clinical importance and 71.02% (201/283) was of subclinical significance (52). A Japanese research study stated the prevalent rates of CM and SCM to be 28 and 13%, respectively, while a closely-related margin of 23.6% was reported from an American study (53).

## Diagnosis of Bovine Mastitis

When the udder of a cow is inflamed, there are several pointers or bio-signatures which are freed and/or influenced as a result of the changes in the milk. These indicators are set out as testing pointers for mastitis (54). Rossi et al. (55) have outlined several testing techniques, ranging from common examinations (somatic cell counts, the California mastitis test) (8) to molecular techniques (polymerase chain reactions) (56), and other sophisticated methods, such as loop-mediated isothermal amplification (LAMP) (57), genetic (58), proteome, as well as transcriptome (59, 60) analyses, nano-assembly and the micro-modeling of compact or mobile gadgets (61) which are used to detect these markers.

### Conventional Screening Techniques for Bovine Mastitis

Regardless of its drawbacks, which include culturing time and costs (62), the microbiological investigation of milk is regarded as the gold standard for detecting bovine mastitis. However, during the inflammatory phase, leukocytes migrate in large numbers to the location site of the inflammation (63). Consequently, the cellularity of the milk can be used to assess the health of the mammary gland of the animal. Techniques including SCC, CMT and Somaticell^®^ are the secondary ways for identifying BM (64).

#### Somatic Cell Count

Somatic cell count (SCC) is a technique that quantitatively detects the various categories of cells in milk (62). Using this method, figures between 100,000 and 272,000 cells mL^−1^ connote the presence of mastitis-causative agents (62). SCC can be carried out using an automated system (Somacount 300 automatic somatic cell counter) or manually by viewing stained microscope slides with appropriate reagents and then viewed using a microscope.

#### The California Mastitis Test

CMT is an elementary, low-cost, quick evaluation that can be conducted within the cattle ranch and is commonly used for identifying subclinical mastitis during milking ((64); 8). The California Mastitis Test is one of the most effective ways to identify mastitis (CMT). To perform the test, only a four-compartmentalized paddle and the CMT reagent are needed. Milk is appropriately collected in the CMT paddle and an equivalent amount of the indicator is added using a horizontal swirling motion for about 30 s. Results are then interpreted accordingly. A strong gel formation that tends to adhere to the paddle and with a leucocyte count of over five million per milliliter of milk sample would point to a strong positive result. A distinct gel formation with a leucocyte count of between 800,000 and 5,000,000,000 mL−1 of milk sample would also indicate a positive result. Distinct precipitate formation with a paddle movement but without gel formation and with a leucocyte count of 400,000 to 1,500,000 per milliliter of milk sample would show a weak positive result. On the other hand, a trace result, denoted by a slight precipitate, which would tend to disappear with paddle movement and which is associated with a leucocyte count of 150,000 to 500,000, would be representative of a mixture without a precipitate and would point to a negative test result (https://extension.missouri.edu/publications/g3653).

#### Somaticell^®^

This is a numerical assessment technique comparable to the Wisconsin Mastitis check that was created with the goal of producing outcomes matching those of the SCC, yet having an edge of portability (65). This technique is also advised for usage in bulk tank milk as a pilot test to determine whether it satisfies the current legal limits. A single-use graded plastic vial with a capped preset scale of SCC is used in this test (63).

#### Automatic Digital Diagnostic

These are newer testing techniques for the examination of mastitis in bovine. They are very easy, fast and field applicable methods (66). These techniques detect the physical, chemical, and biological changes of milk, or quantify the biomarkers in relation to mastitis. The methods include the DeLaval cell counter, the Afimilk mastitis detector, and the Draminski mastitis detector (67). The DeLaval cell counter is suitable for determining the somatic cell count.

Other newer techniques in diagnosing mastitis include the sensor-based system, infra-red thermography (IRT), and proteomic applications. Sensor-based detection systems are usually employed on large farms. A Portuguese study reported the use of magnetic nanoparticles for the actual identification of several staphylococci and specifically *Staphylococcus aureus*. The proteomic approach is helpful in diagnosing, preventing, and forecasting mastitis (68). It aids in the detection of the biomarkers of the intramammary infection (IMI). Hettinga et al. (69) reported the observation of some protein-related substances in relation to the host's reaction to intramammary infection.

### Molecular Techniques for Bacterial Identification for Improved Treatment

Although culture-based approaches for diagnosing intramammary infection (IMI) remain the gold standard in many diagnostic centers, culture-independent methods for identifying bacterial infections in milk have grown more widespread in recent years (8).

When compared to culture-based approaches, molecular-based techniques have been proven to be more sensitive and more rapid since their findings may be ready in only a few hours (8). Bacterial taxonomy and phylogeny are based on conserved gene sequences, particularly the ones that denote ribosomal ribonucleic acids (rRNA). They are used as universal molecular chronometers since they contain sections of varying evolutionary degrees (some extremely conserved; others highly changeable and all-pervasive) (70).

#### Whole Genome Sequencing

This technique yields data on (a) pathogen naming, (b) the characterization of resistant genes (c) epidemiological typing, (d) the selection of peculiar types, regardless of primer development (to track genetic changes in organisms, their adaptations and modifications), and (e) the instant drafting of polymerase chain reaction (PCR) probes based on WGS figures (70). WGS has provided valuable information that has aided advances in present screening techniques, including the detection of the mecC gene (a homolog of the mecA gene that is responsible for methicillin resistance in MRSA) which have elicited the reworking of PCR tests to boost reactivity and circumvent fallacies (71). When prognostics drawn from WGS figures were compared to phenotypic drug vulnerability, WGS was found to be very insightful and specific, as well as to show significant similarities with phenotypic antibacterial susceptibility/resistance approaches (72).

## Conventional Treatment Options for Bovine Mastitis

### Prophylaxis

Bovine mastitis is characterized by the epidemiological triad; host, pathogen, and environment. The expression, “Better prevent than cure,” accurately describes the disease mastitis, as there are many changes, such as harm to the teat and teat canal and udder alveoli that cannot be cured. These are the effects of howl arising from the condition. Mastitis can be circumvented following good acceptable procedures (73, 74). Reduced new infections (NI) and pathogen transmission through improved management standards, the segregation of the animal, and mitigations in the exacerbation of subclinical to clinical mastitis through a regular supply of nutrients are all possible preventative approaches (75). The maintenance of udder health is a process that is always being improved upon and which has already progressed to a high level in that it allows for additional improvements to reduce antimicrobial usage, albeit in a slow and methodical manner (75). Researchers have been working on efficient vaccinations to prevent bovine mastitis for decades, but established vaccines, such as those for *E. coli* or *S. aureus* intramammary infections (IMI) provide only limited protection (76).

### Therapeutic Measures

#### Dry-off Treatment

The dry phase linking two milking periods is critical for teat well-being in dairy cows. It offers an encouraging way to treat current teat contamination, but also provides for conditions where the udder would be likely to incubate a new intramammary infection (IMI). Infections caused by the dry phase often cause increased somatic cell counts (SCC) in early breastfeeding. Management of udder health during the dry season is critical for a smooth transition into the lactation period (75). Epithelial cells that have been damaged or have become senescent are biologically replaced (77). The regeneration and phagocytosis of pathogens cure a substantial amount of IMI without interference, and antimicrobial dry cow therapy is highly effective because the medication is not milked out, thereby allowing for a greater and more consistent concentration of antibiotics in the udder. Thus, most IMIs heal (78).

#### Use of Antibiotics

The goal of managing mastitis with antibiotics is to eradicate the pathogens from the cattle, or worse still, those from the udder. Antibiotics are often used to manage mastitis in lactating cows, as well as to prevent intramammary infections in non-lactating cows (4, 5). More or less 60–70% of the antimicrobials used in cattle are for the prevention and treatment of mastitis (79). Antibiotics, such as the beta-lactams (penicillin G) and macrolides (erythromycin), are generally accepted for the management of bovine mastitis (2, 80). Nevertheless, the non-selective use of antibacterials without the *in-vitro* sensitivity testing of causative organisms is one of the major reasons for failures in the treatment of mastitis (81). Bacterial isolation and antibiotic sensitivity tests are always required for effective antibiotic therapy. According to Holko et al. (82), over 62 percent of the examined mastitis bacterial pathogens were insusceptible to one or more antibacterial drugs. In the majority of instances, the identified microbes were insusceptible to cephalexin, neomycin, penicillin, and streptomycin. Resistance was discovered in 86% of *S. uberis* isolates and 79% of *E. coli* isolates.

##### Mode of Action of Antibiotics

**Figure 2:**
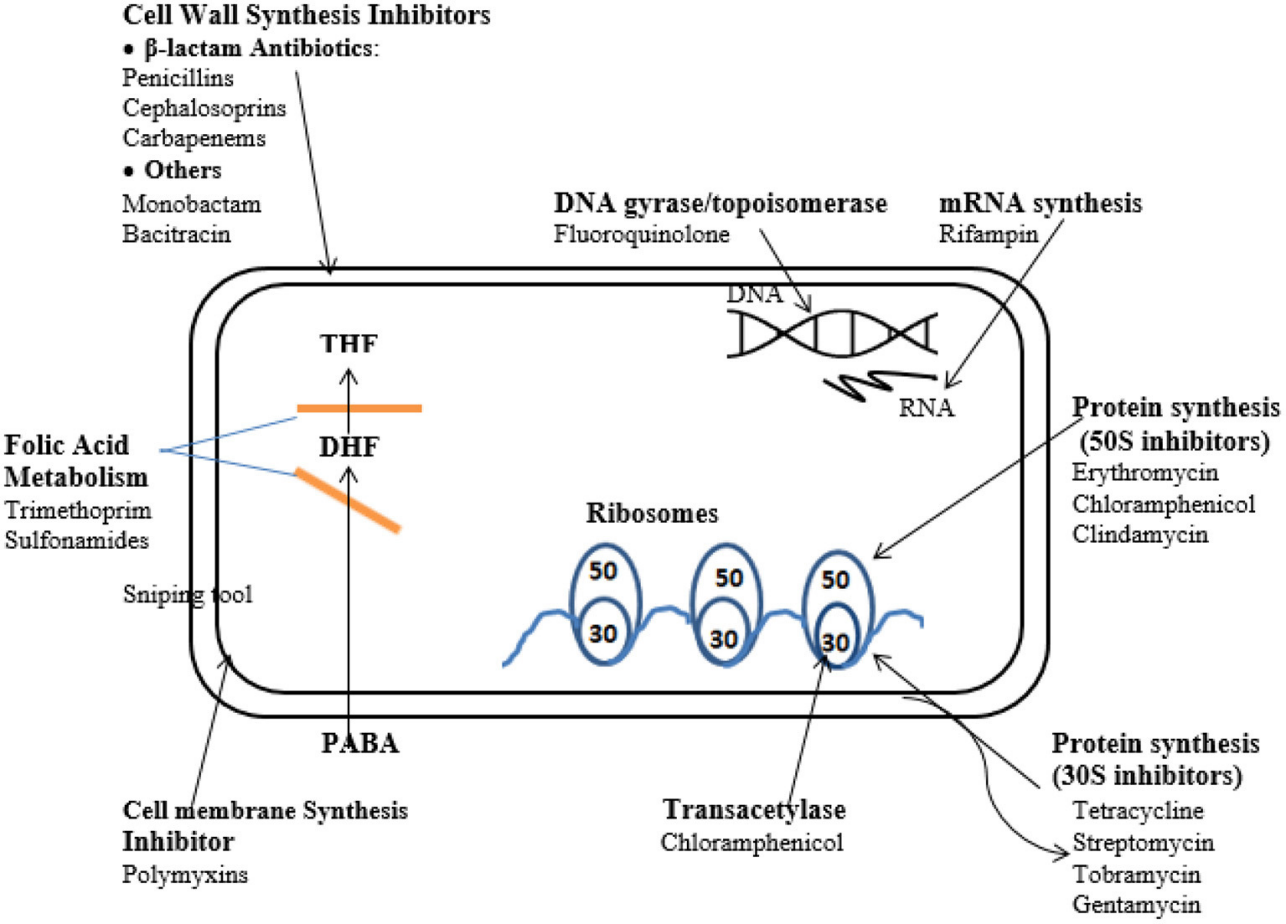
Mode of action of antimicrobial agents.

The course of efficacy of chemotherapeutics can be classified on the basis of the functions that the agents influence which commonly include the inhibition of cell wall synthesis, the suppression of nucleic acid synthesis, the repression of ribosome function, cell membrane function, and folate metabolism inhibition (83) (Figure 2).

##### Development of Antimicrobial Resistance

The overuse of antibiotics in clinical practice has led to bacterial resistance to antimicrobial medicines (84). As it accounts for several losses, MR is a significant challenge in the management of microbial infections in the food and dairy sector and also in the treatment of human diseases. Antibiotic inactivation, target alteration, altered permeability, and the “bypassing” of metabolic pathways are some of the biochemical resistance mechanisms exploited by bacteria (84). The basic anatomy of a Gram-positive bacterium has a cytoplasmic membrane which is surrounded by a thick and stiff mesh, termed the cell wall. Conversely, Gram-negative bacteria have a thin cell wall that is bordered by a second lipid membrane, termed the outer sheath. In Gram-negative bacteria, the sheath is an extra protective layer that inhibits numerous chemicals from entering the organism (84). A growing number of antibiotic-resistant bacteria are jeopardizing the effectiveness of antimicrobials (83). Resistance can be discussed in two broad terms:

✓ **Intrinsic resistance** occurs when microorganisms lack natural antimicrobial target sites and the antibiotic has no effect on them.
✓ **Acquired resistance** occurs when a naturally susceptible bacterium develops a method to resist an antibiotic. Antibiotic resistance in bacteria can be acquired through mutations in regular genetic codes or the uptake of alien insusceptible genetic codes, or both. The presence of an enzyme that causes inactivity in the antibacterial drug, that reduces the uptake of the antimicrobial agent, and the proteinaceous transportation of the anti-infective agent, are all examples of acquired resistance mechanisms (85).

##### Genetics of Antimicrobial Resistance

Bacterial non-susceptibility is a complicated condition that begins with the existence of genes encoding resistance in plasmids or chromosomal genetic material. Considerably aided by the proliferation of flexible genetic features, such as plasmids, transposons, and integrons, there has been a rapid transmission of antibiotic resistance across the various human bacterial groups (86). This is considered to be a matter for serious veterinary concern. Antimicrobial resistance genes (ARGs) have been found to abound on transposable segments (Table 1). Hence, an exclusive occurrence at the genetic level can transmit multiple-drug support traits to a vulnerable receiver. The amplification of integrase genes (intI 1, intI 2 and intI 3) can be used to determine the presence of integrons (98). A couple of researchers have reported some ARGs and their location sites in some isolates of animal origin (Table 1).

**Table 1:** Mode of action, mechanisms of resistance of antibiotics and some antimicrobial resistance genes (ARG) of animal origin.

**Family**	**Mode of action**	**Mechanism of resistance**	**Resistant gene**	**Location^a^**	**Reference**
β-lactams	Cell wall synthesis inhibitors. Binds transpeptidase also known as penicillin binding proteins (PBPs) that help form peptidoglycan	Beta-lactamase production primarily-*bla* genes	*mec*C (mecALGA251) *mec*A *bla*Z	C C	(83, 86, 87)
	Inactivates the enzyme; beta-lactamase	Expression of alternative PBPs		Tn, P, C	
β-lactamase inhibitors	Hydrolysis of the beta-lactam ring	Production of extended spectrum beta-lactamases (ESBLs)	*bla*_SHV_ *bla*_TEM_	P, C P, C	
Fluoroquinolones	Binds DNA-gyrase or topoisomerase II and topoisomerase IV; enzymes needed for supercoiling, replication and separation of circular bacterial DNA.	Target modification Decreased membrane permeability Efflux pumps	*qnr*A *qnr*B *qnr*S 9, 10	P P P	(85, 88)
Macrolides, Licosamides and Streptogamin (MLS)	Binds the bacterial 50 S ribosomal subunits; inhibit protein synthesis	Target site modification	*erm*A, (*erm*B), *erm*C, *erm*F, *erm*T*, erm* (73) *erm* (83)	C, Tn, P, (Tn,P) P, C P, P C	(89, 90)
		Active drug efflux	*msr* (A) except lincosamides	C, P	
Aminoglycosides	Bind to the bacterial 30 S ribosomal subunit thus inhibit bacterial protein synthesis	Target site modification [*via* the action of 16 S rRNA methyltransferases (RMTs)]			(89, 91, 92)
		Enzymatic drug modification (adenylation, acetylation and phosphorylation)	aacA-aphD *aad*D	Tn, P, C P, C	
		Efflux systems	*aad*E *str*	Tn, P P	
Tetracyclines	Bind reversibly to the 30 S ribosomal subunit as such blocks the binding of the aminoacyl-tRNA to the acceptor site on the mRNA-ribosome complex	Efflux systems Target modification Inactivating enzymes Ribosomal protection	*Tet* (K), *tet* (K) *Tet* (M), *tet* (O)	P Tn/C, C	(93, 94)
Sulfonamides (Folate pathway inhibitors)	Inhibit the bacterial enzyme dihydropteroate synthetase (DPS) in the folic acid pathway, thereby blocking bacterial nucleic acid synthesis	Excessive bacterial production of dihydrofolate reductase (DHFR) Reduction in the ability of the drug to penetrate the bacterial cell wall Production of altered forms of the dihydropteroate synthetase (DPS) enzyme with a lower affinity for sulfonamides Hyperproduction of PABA, which overcomes the competitive substitution of the sulfonamides	*sul*1 *sul*2 *sul*3	P, C	(95–97)

a *C, chromosomal DNA; P, plasmid; Tn, transposon*.

##### Challenges of Treatment With Antibiotics

The shortcomings of this strategy include low cure rates, the increased occurrence of antibiotic-resistance; pressing public health concerns, reduced food safety, security, and their upshots (12, 13), as well as the appearance of flakes in cow products (5, 11, 99). Milk tainted with antibiotics can cause sensitive reactions, alterations in the microbial makeup of the gut, and the growth of antibiotic-resistant strains in the general public (100). Because antibiotics aren't allowed to reach them, bacteria that can live intracellularly within the udder and that cause sores to develop are laborious to treat. This is evident with *S. aureus*, where the cure rate, based on the generally accepted antibiotics (e.g., pirlimycin), is about 10–30% (76). Consequently, these pathogens have developed resistance to antibiotic treatment. Thus, the World Health Organization (WHO) has released guidelines for limiting antibiotic use in livestock farming (101, 102).

More so, from the one-health perspective, mastitis-causing bacteria have broken through a number of hierarchical barriers, allowing for zoonotic transmission from bovines to people *via* milk and meat, either locally or remotely, and as a result, disease control has been quite challenging (103).

## Alternative Treatment to Conventional Antibiotics

### Ethno-Veterinary Medicine

Ethno-veterinary medicine (EVM) is the application of humankind's expertise, abilities, practices, habits, and credence in managing and safekeeping the well-being of their animals (104). These hands-on skills are shared or transferred from age to age only through the oral medium (105). Both the scientific and consumer communities are increasingly turning to herbal plants as health promoters (106). In the interests of the foremost availability of these products and the cheap manufacturing costs around these items, studies using local plants must of necessity encompass multiple geographical locations. Kalayou et al. (28) investigated the antibacterial potency of several plant species against mastitis, with Croton aurea, Croton macrostachyus, Achyranthes aspera, Nicotiana tabacum and *Vernonia* species producing the most promising effects of all tested plants. Findings by Serunkuma et al. (107) established the efficacy of the acetone extract of the *Acacia nilotica* bark and the *Tetradenia riparia* flower against bacterial species cultured from mastitis samples from a farm. Across the board, the development of *S. agalactiae, S. uberis, E. coli* and *S. aureus* was suppressed by a methanol extract derived from *Spathodea campanulata* (108). Globally, the use of diverse plants from distinct geographical terrains to treat livestock infections remains common (Table 2). *In-vitro* assays into medicinal plants reveal their potency as antibacterial, anti-inflammatory or immune-modulatory agents (109).

**Table 2:** Ethno-veterinary medicinal plants used in the dairy and livestock farming.

**Scientific name**	**Family**	**Life form**	**Plant part used**	**Animal**	**Use/disease**	**Reference**
*Erysimum* melicentae Dunn.	Brassicaceae	Herb	Whole plant	Cattle and sheep	For general health improvement	(15)
Becium obovatum (E. Mey. Ex Benth. In E. Mey.) N.E. Br.	Lamiaceae	Herb	Root	Livestock	Mastitis, Black leg, listeriosis/encircling disease, diarrhea	(16)
*Malva parviflora*	Malvaceae	Herb	Whole Plant	Cattle	Mastitis	(17)
*Brucea antidysenterica*	Simaroubaceae	Tree	Leaf	Cattle	Mastitis	(18)
*Acorus calamus* L.	Acoraceae	Herb	Rhizome	Cows, Sheeps, Goats, Donkeys, Camels, Buffaloes	Mastitis, Anaplasmosis, constipation, heal wounds, dysentery, body tonic, gastric problems, bloating, indigestion, urinary disorder	(19)
*Prosopis juliflora* (Sw.) DC.	Mimosaceae	Shrub	Leaf	Cattle	Infections	(20)
*Triticum* sp.	Poaceae	Herb	Aerial parts, Bran	Livestock	Mastitis, breast lumps, difficulty of birth, retained placenta, increasing egg production	(21)
*Arachis hypogea* L.	Fabaceae	Shrub	Seed and seed oil	Goat and cattle	Increased milk production	(22)
*Peganum harmala* L.	Zygophyllaceae	Herb	Leaf, branches	Buffalo, Cattle, Dog	Mastitis	(23)
*Citrus limon* (L.) Osbeck	Rutaceae	Tree	Fruit	Buffalo, Cattle, Goat	Mastitis	(23)
*Withania somnifera* (L.) Dunal	Solanaceae	Shrub	Root	Buffalo, Cattle, Goat	Mastitis	(23)

#### Application of Ethno-Veterinary Medicine in the Treatment and Management of Mastitis

Ethno-veterinary medicine is a local animal healthcare system that incorporates traditional beliefs, knowledge, skills, methods, and practices. It includes the traditional treatment of veterinary illnesses, as well as the spiritual aspects of the treatment (24). Depending on the active substances to be extracted, the delivery route, and the medicinal purpose (prophylaxis or therapeutics), the method used to make ethno-veterinary medications differs. Infusions, decoctions, powders, drips, fumes, pastes, and ointments are made from medical plants, animal products, minerals, and other inorganic ingredients accessed by livestock owners and ranchers. These might be treated topically with drenches, or intra-nasally with smoke, vaccines, or suppositories, vapors, or massages (24).

Abbasi et al. (23) reported that paste made from the fruit juice of *Citrus limon* and sugar fed to animals and applied topically to the mammary glands for 10–15 days is used to treat mastitis in buffalo, cattle and goats. Also, the application of a paste of 200 g of fresh crushed roots of *Withania somnifera* to the udder of a cow and goat respectively for up to a week successfully treats the diseased condition. It is also noteworthy to state that the preparation and application of the smoke of the leaves and branches of *Peganum harmala* over a period of about 5 days successfully treats mastitis in cattle and horses. The anti-inflammatory effects of the topical application of the extract of fresh leaves of *Rumex nepalensis* to the affected part for about 5 days has also been reported. A poultice of young twigs of *Calotropis procera* applied to a swollen udder also relieves pain and inflammation (23). The treatment of BM using several of the EVM currently available is highlighted below (Table 3).

**Table 3:** Ethno-veterinary medicinal products, preparation and administration for the treatment and control of mastitis.

**Product**	**Dose rate**	**Indication**	**Reference**
Kali mur 6x + Calc Flur 6x	B.I.D. for 1 week to 10 days	Presence of clots in milk	(24)
Calundula Q + Belladonna 30+ Dulcamara Q + Echinaea 30 aa 1 ml Made upto 20 ml with distilled water	10 ml B.I.D. intra mammary injection for 2 to 3 days. Massage the udder to disperse the medicine uniformly	Inflammation of the udder with loss of appetite, fever congestion and injury	(24)
Silicea 1M + Calc. Sulph 200	Q.I.D. for 2–7 days	Mastitis without anorexia, udder is hard and with clots	60
Phytolocca 200 + Calc. Fluor 200 + Silicea 30 + Belladonna 30 + Arnica 30 + Conium 30 + Ipeca 30 aa 0.5 ml. Made up to 30 ml vimeral	B.I.D. for 2–4 days	In acute, subacute and chronic mastitis.	(24)
Kali Mur 30	Q.I.D. for 2–5 days	Mastitis without anorexia, hardness of udder and white or gray or cream color clots	(24)
Ferrum Phos 6x	Q.I.D. for 2–5 days	Mastitis without anorexia, blood in milk with or without bad smell	(24)
Plant	Plant part	Administration/dosage for cows	Reference
*Allium sativum* L.	Rhizome	250 g, grinded with butter and administered orally for 7 days	(25–27)
*Allium cepa* L.	Bulb	Heated in oil, given as food supplement once per day during 2 or 3 days or until the animal gets better (topical application and vaginal washes)	(27)
*Asphodelus tenuifolius* Cav.	Aerial part	Heated with barley peels (topical application)	(27)
*Amomum subulatum* Roxb.	Fruit	25 g, given orally for 3 days.	(25)
*Brassica compestres + Curcuma longa*	Seeds + root	250 g seeds are grinded with 50 g root and administered orally for 5 days	(25)
*Brucea antidysenterica* JF. Mill	Seed	Add 1 L of water to the ground fresh seed given orally once per day for 3 days	(26)
*Peganum harmala + Triticum sativum*	Fruit + stem crushing (hay)	50 g + 2 Kg, fumigation of harmal by putting it on fired hay under the affected udder for 4 days	(25)
*Capsicum annuum*	Fruit/whole plant	50 g, given orally for 8 days	(25)
*Sesamum indicum*	Seed oil	250 ml, mixed oil in 1.5 L of milk whey, and given orally for 7 days	(25)
*Citrus limon*	Extract	With raw sugar given orally for 5 days	(25)
*Osyris quadripartita* Decn.	Root	Pound the fresh root and mix with water, filter and administered orally for 6–7 days, daily	(26)
*Gossypium hirsutum* L.	Flowers	250 g, boiled in 1 L water to 250 ml, then drenched for 3 days	(25)
*Galium aparine* L.	Vine	500 g, given as decoction drench for 3 days	105
*Chenopodium ambrosioides* L.	Leaf	After grinding the fresh leaf, mix with water to prepare (liquid) 1 L then it is given orally once	(26)
*Solanum* spp.	Leaf	The fresh leaf and root are chewed by the local healer and spit to the mouth of the animal for 2 days	(26)
*Artemisia herba-alba* Asso	Aerial part	Heated with barley peels and/or aggaya (topical application)	107
*Ricinus communis* L.	Leaf	Pound about 50 g of fresh leaf and mix with 1 L of water then administered orally 1 L/day (every morning) for 2 days	(25, 26)
*Cynomorium coccineum* L.	Whole plant	Washes with decoction water	(27)
*Hordeum* vulgare L.	Seed	Roasted seeds mixed with water	(27)
*Ziziphus spina-christi*	Leaf	The leaves are ground and applied on the affected teat quarter	(28)
*Achyranthes aspera* + *Commicarpus podunculosus*	Root + leaf	The fresh root of an A*chyranthes* aspera is chopped and bounded together with a leaf of *Commicarpus podunculosus*. This will be mixed with water and given orally	(28)

An herbal mixture of *Aloe vera* (L.) Burm. -F., *Curcuma longa* L. and calcium hydroxide, as documented by a traditional healer, was reported to be efficient against BM causing pathogens (110). Since the components of the mixture had a synergistic effect, their activity against the clinical mastitis pathogens was successful. The formulation was found to be antimicrobial and anti-inflammatory and to have wound cleansing and healing properties.

#### Natural Products (Plants): the Sleeping Giant of the Modern Pharmaceuticals

**Figure 3:**
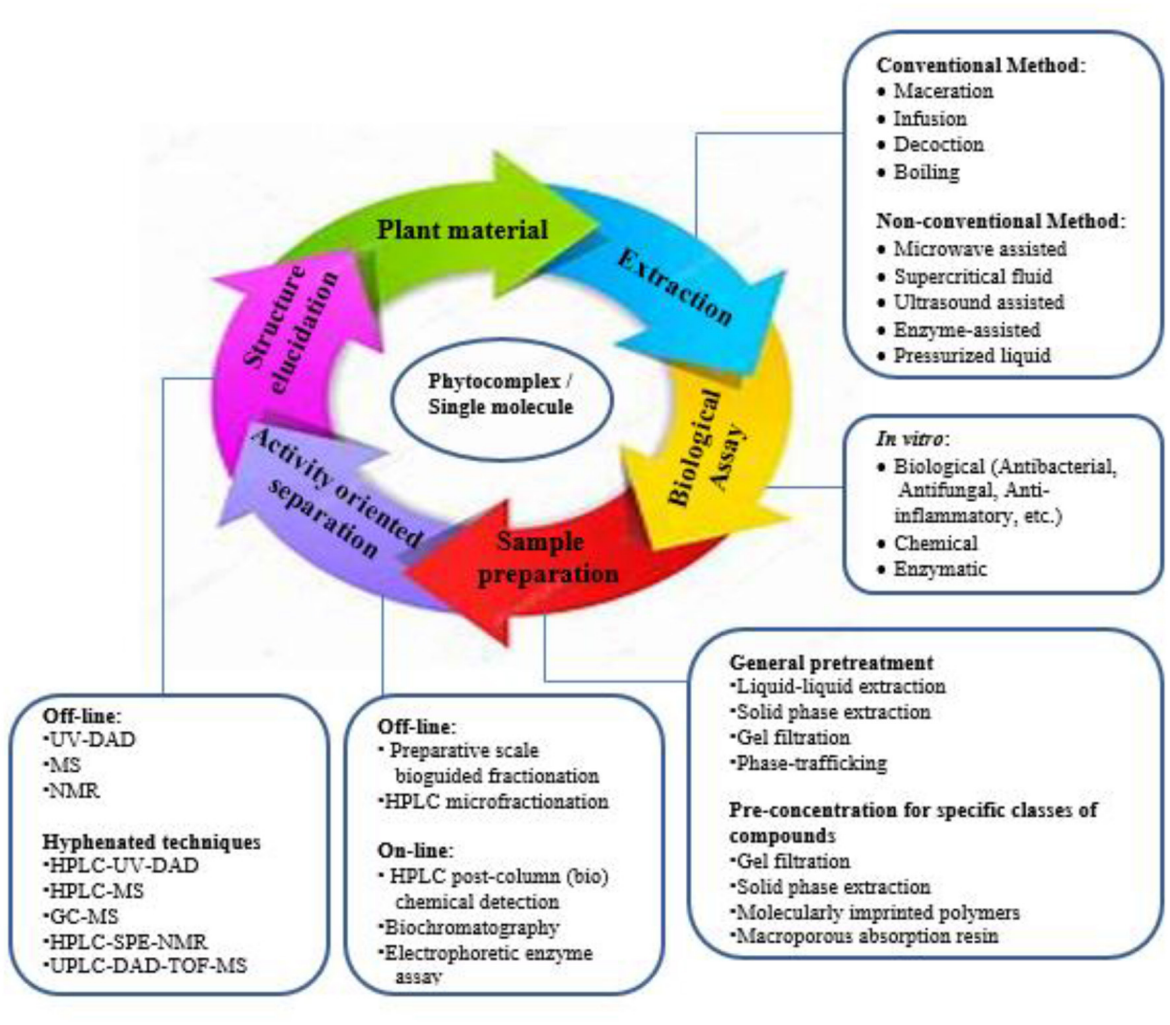
Ethno-pharmacological methodologies.

Globally, the plant domain renders a diversity of species utilized as treatments for various ailments (111). The World Health Organization (WHO) report states that traditional medicines, including those used for plant decoctions or functional compounds are used by a high percentage of the population of the world (112). An understanding of the plant parts used historically as treatments is the most essential requirement in the ethno-pharmacological approach (Figure 3). Chinese herbal medicine and Ayurveda are the prominent traditional medicines used in treating various diseases, but where no texts are available, the ethno-botanical survey is the only way in which to learn about the traditional uses of medicinal plants.

Natural products and/or organic by-product forms continue to play a pivotal role in the medication upshot process. As a result, biological diversity offers an endless supply of new chemical entities (NCEs) with their potential as therapeutic leads. These NCEs are derived productsgenerated by plants to shield them from herbivores and pathogens, or to attract pollinators (111).

The efficacy of secondary metabolites in plants is an indication of the strength of natural products as potent modern pharmaceuticals (113, 114). Researchers, including Smyth et al. (115) and Wu et al. (116), have issued reports on sample preparation and characterization. However, these reviews primarily focus on a chemosystematics-disposal method: the plant type chosen for testing is known to contain specific derivatives (amino acids, alkaloids and steroids); hence, the best extraction/purification/separation of these compounds is achieved by using the most appropriate extraction methodology and analytical technique (111, 117).

The antimicrobial peptides (AMPs) of plant origin are a source of biocontrol agents against bacteria, fungi, nematodes, insects, and pests (118). They are classified into different groups to include the type of charge, the disulfide bonds present, the cyclic structure, and the mechanism of action. The common types include cyclotide, defensins, hevein-like proteins, knotin-type proteins, lipid-transfer proteins, protease inhibitors, snakins, and thionins (119). Researchers have reported the isolation and identification of peptides, defensins, protease inhibitors, lectins, thionin-like peptides, and vicilin-like peptides from solanaceae (120, 121). Particularly peptides and peptide-rich extracts of plants have been found to promote antibacterial activity against various strains of bacteria (121). Although their mechanism of action remains unclear, their antibacterial activity could be attributed to changes in membrane permeability (119).

It is clear that peptide contact with the cell membrane induces changes in the structure and aggregation state of the peptides which are adapted by membrane lipids *via* changes in their lipid conformation and packing structure (122). Given that the outer membranes of Gram-negative bacteria or the cell walls of Gram-positive bacteria have negatively-charged surfaces, no fundamental difference was found in the way in which AMPs worked on them. Moreover, the cell wall of Gram-positive bacteria have pores (40 to 80 nm) through which multiple AMPs may readily pass to interact with the target location (123).

#### Characterization of Secondary Metabolites in Plants

Plants produce an extensive array of chemical molecules including those that are indirectly involved with development and growth and which are termed secondary metabolites (124). The polar difference of the fragment and the solvent utilized determine the extraction procedure for bioactive substances. More so, depending on the qualities of each biomolecule of interest, plant-derived particles from unrefined extracts can be additionally segregated, isolated, and refined using a mix of separation procedures and various approaches (125). Several of such techniques are presented below:

##### High Performance-Thin Layer Chromatography

HPTLC is a sophisticated form of TLC which yields superior separation efficiency results. For fingerprint analysis, TLC is the most frequent method. Capillary action pulls the energetic section into the immobilized part. The polarity of each component is used to distinguish samples. The HPTLC characterization is mostly carried out to investigate substances with small to medium polar differences. The approach is generally used in the process upshot, the observation of impurities in the herbal by-products, the identification of the pesticide, as well as its content, and in a standard check of herbal mixtures and wellness foods (126).

Unrefined extracts are used in conjunction with standard molecules, and software is available to assess the quantities of chemicals available in the test material (125).

##### High Performance Liquid Chromatography

Previously known as high pressure liquid chromatography, this is a more sophisticated method than HPTLC. A liquid sample is injected into a stream of solvent (mobile phase), and passes through a separation medium-filled column (stationary phase). As the sample components pass through the column, a process called differential migration separates them (127). The stationary phase usually involves a column filled with minute porous matter, with the liquid vigorous part being injected *via* the column. Currently, the most effective technology is a merger of LC/MS and HPLC (128).

##### Ultra-Performance Liquid Chromatography

This is a comparably recent approach in liquid chromatography that opens up new possibilities, particularly in terms of time and solvent consumption. This technique takes total advantage of chromatographic principles to stimulate separations using columns chocked with smaller particles and/or higher flow rates for faster processing with extraordinary resolution and sensitivity (129). The UPLC is based on the premise of using a stationary phase with particles smaller than two (2) μm in diameter (129).

##### Liquid Chromatography-Mass Spectroscopy

LC–MS is an analytical chemistry approach that merges the actual dissociation potential of liquid separation (or HPLC) with the aggregate scrutiny potentials of mass spectrometry (MS). The advantages of LC-MS are similar to those of GC-MS: excellent selectivity, as well as the capacity to work with complex compounds (125). The ability to multiplex LC-MS tests, allowing for the measurement of numerous medicines and metabolites in a single run, is a beneficial feature of these assays. When compared to GC-MS screening, the use of LC-tandem MS for toxicology screening is appealing since it has the ability to provide more confidence in the identification process and also to identify a wider spectrum of medicines, toxins, and their metabolites, and to simplify sample preparation (130).

##### Nuclear Magnetic Resonance

Nuclear Magnetic Resonance (NMR) is a nuclei (nuclear)-specific spectroscopy with numerous applications in the physical sciences and industry. The inherent angular momentum characteristics of atomic nuclei are investigated using nuclear magnetic resonance, which employs a massive magnet (magnetic). Similar to other spectroscopic techniques, NMR relies on ionizing emission elements (audio waves) to ease transference between degrees of atomic powers (131). NMR is mostly used to check for the structure of fragments. The combination of Mass Spectrometry, NMR and Liquid Chromatography (LC–NMR–MS) provides metabolic organizational information about innovative pharmaceuticals in production, as well as their regular uses (125). The speed and sensitivity of NMR detection have recently been improved, and have been proven to be effective in pharmacokinetics, drug metabolism and development and toxicity research (132).

##### Gas Chromatography-Mass Spectroscopy

GC-MS is frequently used to test the purity and stability of organic compounds, as well as to distinguish, both qualitatively and quantitatively, between the constituents of a mixture. The preparation of the sample, loading, and dissociation on a GC apparatus are the first steps in a GC-MS experiment. MS is the GC detector and it generates a chromatogram that indicates the amount of each chemical in relation to its retention duration (133). Its application is employed in a variety of spheres inclusive of environmental chemistry for studies on water, soil and the atmosphere, as well as in forensic science for drug detection (or metabolites) well as in forensic science for drug (134).

##### Infrared Spectroscopy

Infrared spectroscopy (IR spectroscopy or vibrational spectroscopy) studies the interplay between infrared radiation and different materials through absorption, emission, or reflection. It evaluates mixtures in various forms; solid, liquid, or gaseous. A sample's infrared spectrum is recorded by passing an infrared light beam through it. Absorption occurs when the IR frequency is similar to the frequency of vibration of (a) bond(s). The amount of energy absorbed at each frequency can be determined by examining the transmitted light (or wavelength). A monochromator can be used to scan the wavelength range and to make this measurement. Alternatively, a Fourier transform (FTIR) device can be used to measure the complete wavelength range, and then a dedicated process can be used to build a transmittance or absorbance spectrum. Other methods in molecular vibrational spectroscopy include Raman spectroscopy, high resolution electron energy loss spectroscopy (HREELS), and electron energy loss spectroscopy (EELS) (135).

## Conclusion and Future Perspectives

Ethno-veterinary practices have been identified and evaluated as an upshot in lowering the use of antibiotics and other veterinary medicines in dairy farming. The application of people's intellects, skills, techniques, traditions and faith is the domain of ethno-veterinary medicine. Thus, these skills need to be properly documented as they are shared or transferred from age to age only through the oral medium. Given the increasing interest in herbal medicines, sustainability programmes should be put in place to avoid the threats to these plants and their possible extinction. Proper investigations involving *in vivo* studies into the aforementioned are critical to the success of the war being waged against antimicrobial resistance in the dairy industry. Hence, it is imperative that the ethno-pharmacology of medicinal plants should be exploited to optimize their valuable use as antibacterial, anti-inflammatory and immune-modulatory agents and antioxidants.

## Author Contributions

CA: conceptualization and resources. DA, BO, TA, JF, KM, and CA: writing–review and editing. DA: writing–original draft preparation. CA, AA, and OF: supervision and editing. All authors contributed to the article and approved the submitted version.

## Conflict of Interest

The authors declare that the research was conducted in the absence of any commercial or financial relationships that could be construed as a potential conflict of interest.

## Publisher's Note

All claims expressed in this article are solely those of the authors and do not necessarily represent those of their affiliated organizations, or those of the publisher, the editors and the reviewers. Any product that may be evaluated in this article, or claim that may be made by its manufacturer, is not guaranteed or endorsed by the publisher.
